# DNA-triggered AIM2 condensation orchestrates immune activation and regulation

**DOI:** 10.1093/procel/pwag024

**Published:** 2026-03-25

**Authors:** Quanjin Li, Xiaohan Geng, Huiwen Yan, Zhaolong Li, Miao Shi, Ziqi Zhu, Tongxin Niu, Chunqiu Zhao, Kaile Shu, Yina Gao, Han Feng, Songqing Liu, Qiuyao Jiang, Pengcheng Bu, Dong Li, Pu Gao

**Affiliations:** School of Life Science, Beijing Institute of Technology, Beijing 100081, China; National Laboratory of Biomacromolecules, CAS Center for Excellence in Biomacromolecules, Institute of Biophysics, Chinese Academy of Sciences, Beijing 100101, China; National Laboratory of Biomacromolecules, CAS Center for Excellence in Biomacromolecules, Institute of Biophysics, Chinese Academy of Sciences, Beijing 100101, China; University of Chinese Academy of Sciences, Beijing 100049, China; State Key Laboratory of Epigenetic Regulation and Intervention, Institute of Biophysics, Chinese Academy of Sciences, Beijing 100101, China; National Laboratory of Biomacromolecules, CAS Center for Excellence in Biomacromolecules, Institute of Biophysics, Chinese Academy of Sciences, Beijing 100101, China; University of Chinese Academy of Sciences, Beijing 100049, China; National Laboratory of Biomacromolecules, CAS Center for Excellence in Biomacromolecules, Institute of Biophysics, Chinese Academy of Sciences, Beijing 100101, China; Medical Science and Technology Innovation Center, Shandong First Medical University & Shandong Academy of Medical Sciences, Jinan 250000, China; National Laboratory of Biomacromolecules, CAS Center for Excellence in Biomacromolecules, Institute of Biophysics, Chinese Academy of Sciences, Beijing 100101, China; University of Chinese Academy of Sciences, Beijing 100049, China; Center for Biological Imaging, Institute of Biophysics, Chinese Academy of Sciences, Beijing 100101, China; National Laboratory of Biomacromolecules, CAS Center for Excellence in Biomacromolecules, Institute of Biophysics, Chinese Academy of Sciences, Beijing 100101, China; University of Chinese Academy of Sciences, Beijing 100049, China; University of Chinese Academy of Sciences, Beijing 100049, China; State Key Laboratory of Epigenetic Regulation and Intervention, Institute of Biophysics, Chinese Academy of Sciences, Beijing 100101, China; National Laboratory of Biomacromolecules, CAS Center for Excellence in Biomacromolecules, Institute of Biophysics, Chinese Academy of Sciences, Beijing 100101, China; National Laboratory of Biomacromolecules, CAS Center for Excellence in Biomacromolecules, Institute of Biophysics, Chinese Academy of Sciences, Beijing 100101, China; National Laboratory of Biomacromolecules, CAS Center for Excellence in Biomacromolecules, Institute of Biophysics, Chinese Academy of Sciences, Beijing 100101, China; Medical Science and Technology Innovation Center, Shandong First Medical University & Shandong Academy of Medical Sciences, Jinan 250000, China; University of Chinese Academy of Sciences, Beijing 100049, China; State Key Laboratory of Epigenetic Regulation and Intervention, Institute of Biophysics, Chinese Academy of Sciences, Beijing 100101, China; National Laboratory of Biomacromolecules, CAS Center for Excellence in Biomacromolecules, Institute of Biophysics, Chinese Academy of Sciences, Beijing 100101, China; University of Chinese Academy of Sciences, Beijing 100049, China; State Key Laboratory of Membrane Biology, Tsinghua-Peking Center for Life Sciences, Beijing Frontier Research Center for Biological Structure, IDG/McGovern Institute for Brain Research, New Cornerstone Science Laboratory, School of Life Sciences, Tsinghua University, Beijing 100084, China; National Laboratory of Biomacromolecules, CAS Center for Excellence in Biomacromolecules, Institute of Biophysics, Chinese Academy of Sciences, Beijing 100101, China; University of Chinese Academy of Sciences, Beijing 100049, China; Medical Science and Technology Innovation Center, Shandong First Medical University & Shandong Academy of Medical Sciences, Jinan 250000, China

**Keywords:** AIM2, phase separation, innate immune, inflammasome, PANoptosome

## Abstract

The innate immune sensor AIM2 detects cytosolic DNA and initiates inflammatory responses, yet its activation mechanism remains incompletely understood. Here, we show that AIM2 undergoes liquid–liquid phase separation upon DNA binding, forming dynamic condensates both *in vitro* and in cells. These condensates serve as platforms for inflammasome and PANoptosome assembly, promoting immune activation across multiple pathways. Direct structural determination from condensates reveals the assembly of active-form ASC filaments. Mechanistically, liquid-phase condensation is governed by multivalent interactions involving different AIM2 domains, including previously uncharacterized regions and species-specific elements. *In vitro* and *in vivo* assays show that mutants specifically disrupting condensation impair immune complex assembly, cell death initiation, antimicrobial defense, and intestinal homeostasis. Moreover, AIM2-DNA condensates function as regulatory hubs targeted by host- and pathogen-derived factors to balance immune homeostasis or facilitate immune evasion. These findings establish liquid-phase condensation as a fundamental mechanism of AIM2 activation and a potential therapeutic target.

## Introduction

Aberrant DNA resulting from pathogen infection or cellular stress serves as a critical pathogen- or damage-associated molecular pattern (PAMP or DAMP), which is detected by pattern recognition receptors (PRRs) to initiate innate immune responses. Among these PRRs, absent in melanoma 2 (AIM2) plays a crucial role in recognizing cytosolic double-stranded DNA (dsDNA) and recruiting the adaptor ASC and protease caspase-1 to form an inflammasome (Bürckstümmer et al., 2009; Fernandes-Alnemri et al., 2009; Hornung et al., 2009; Lu et al., 2014b; Roberts et al., 2009). Inflammasome assembly induces autoprocessing of caspase-1, which subsequently cleaves downstream substrates gasdermin D (GSDMD) and proinflammatory cytokines pro-IL-1β and pro-IL-18, driving pyroptosis and inflammation (Broz and Dixit, 2016; Ding et al., 2016; Kayagaki et al., 2015; Liu et al., 2016; Man and Kanneganti, 2015; Martinon et al., 2002; Shi et al., 2015, 2017; Xia et al., 2021). Beyond canonical inflammasome signaling, AIM2 also assembles with proteins such as ZBP1, RIPK1, RIPK3, FADD, and caspase-8 to form the AIM2 PANoptosome, a multi-protein complex that mediates inflammatory cell death (PANoptosis) by integrating pyroptotic, apoptotic, and necroptotic pathways (Lee et al., 2021; Malireddi et al., 2019; Oh et al., 2023; Sagulenko et al., 2013). AIM2-mediated immune responses play vital roles in infection defense, tumor suppression, and neurodevelopment (Fernandes-Alnemri et al., 2010; Jones et al., 2010; Kumari et al., 2020; Lammert et al., 2020; Man et al., 2015a, 2015b; Rathinam et al., 2010; Sauer et al., 2010; Warren et al., 2010). However, its aberrant activation is associated with autoimmune and cardiovascular diseases, such as systemic lupus erythematosus and atherosclerosis (Cao et al., 2024; Du et al., 2022; Fidler et al., 2021; Kumari et al., 2020; Man et al., 2015a; Sharma et al., 2019). Thus, precise regulation of AIM2 is essential for maintaining immune homeostasis. Host proteins like p202 act as negative regulators of AIM2 inflammasome (Roberts et al., 2009; Ru et al., 2013; Yin et al., 2013); while pathogens, such as HSV-1, encode inhibitors like VP22 to suppress AIM2 activation and evade immune detection (Maruzuru et al., 2018, 2021).

AIM2 comprises an N-terminal pyrin domain (PYD) for homotypic oligomerization, a C-terminal HIN domain for DNA binding, and a flexible intrinsically disordered region (IDR) connecting them. *In vitro* structural and biochemical studies suggest a potential activation model where multiple AIM2 molecules bind to the same dsDNA strand via their HIN domains, with peripheral PYD domains oligomerizing into a filamentous nucleation site that promotes ASC assembly and inflammasome formation (Garg et al., 2023; Jin et al., 2012, 2013; Lu et al., 2014a, 2014b, 2015; Morrone et al., 2015). However, current understanding does not fully explain many observations and functional aspects of AIM2 activation. It is unclear how relatively low levels of AIM2 efficiently and simultaneously converge on a focused dsDNA region within the vast cytosolic space. The formation of the nucleation site requires the assembly of adjacent PYDs, while their corresponding HIN domains occupy only ∼20 base pairs (bp) of DNA, which is far shorter than the >80 bp needed for minimal AIM2 activation and the >300 bp required for optimal activation (Garg et al., 2023; Jin et al., 2012; Lu et al., 2015; Morrone et al., 2015). While the model focuses on HIN-DNA and PYD-PYD interactions, cancer-associated mutations in the IDR and other “non-essential” regions imply that these areas may also contribute to AIM2 function (Fig. S1A). Furthermore, the current simplistic interaction model does not explain how AIM2 coordinates the recruitment of multiple PANoptosome components and how its activity is regulated by various host- and pathogen-derived factors. These discrepancies highlight important gaps in our understanding of AIM2 activation and regulation, underscoring the necessity for additional in-depth mechanistic insights.

## Results

### DNA induces AIM2 to form liquid-phase condensation *in vitro* and in cells

**Figure 1.**
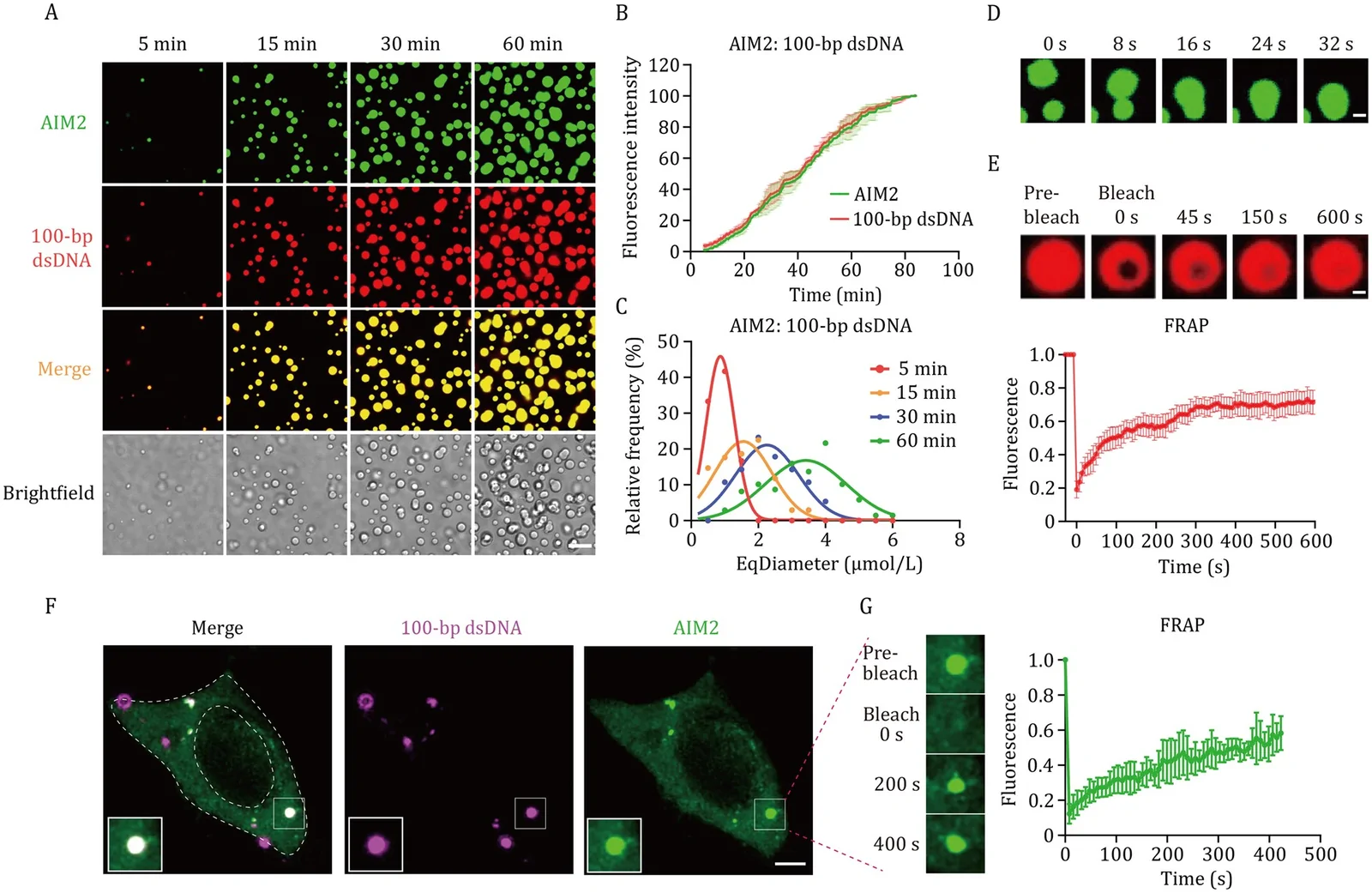
**dsDNA induces AIM2 to form liquid-phase condensation *in vitro* and in cells**. (A) Time-lapse images of droplets formed by 5 μmol/L AF488-labeled mAIM2 and 1 μmol/L cy3-labeled 100-bp dsDNA in 300 mmol/L NaCl buffer. Scale bar, 10 μm. (B) Fluorescence intensities of mAIM2-DNA droplets formed over the time course of 80 min. The maximal fluorescence intensity was normalized to 100%. Values shown are means ± SD. *n* = 3 images. (C) EqDiameter frequency distribution of mAIM2-DNA droplets that formed as described in (A) at the indicated time points. (D) Time-lapse micrographs of merging mAIM2-DNA droplets that formed as described in (A). Scale bar, 1 μm. (E) FRAP analysis of mAIM2-DNA droplets that formed as described in (A). Top panel: time-lapse images of mAIM2-DNA droplets before and after photobleaching. scale bar, 1 μm. Bottom panel: quantification of FRAP data. Values shown are means ± SD. *n* = 3 condensates. (F) Representative live-cell images of mAIM2-DNA puncta in HEK293T cells expressing GFP-mAIM2 (Tet-on) at 5 h after transfecting with 1 μg/mL cy5-labeled 100-bp dsDNA and treating 25 µg/mL doxycycline to induce the expression of GFP-mAIM2. The mAIM2-DNA puncta highlighted by the white box are magnified at the bottom left. scale bar, 4 μm; 4 μm for magnified images. (G) FRAP analysis of mAIM2-DNA puncta, as shown in (F). Left panel: time-lapse images of mAIM2-DNA puncta before and after photobleaching. 4 μm for magnified images. Right panel: quantification of FRAP data. Values shown are means ± SD. *n* = 3 puncta.

Upon stimulation with DNA or pathogen, endogenous mouse (m) and human (h) AIM2 robustly form puncta with DNA and ASC in mouse bone marrow-derived macrophages (BMDMs) and human monocytic THP-1 cells (Fig. S1B and S1C). We reasoned that elucidating the characteristics and assembly mechanisms of AIM2 puncta could provide critical insights into AIM2-mediated signaling activation. To explore the properties of AIM2 puncta *in vitro*, we expressed and purified full-length mAIM2 and hAIM2 with an MBP tag. Upon mixing with 100-bp dsDNA and treatment with TEV protease to remove the MBP tag, mAIM2 rapidly forms liquid-like droplets with dsDNA (Figs. 1A and S1D). As time progressed, mAIM2-DNA droplets fuse with each other into larger ones, accompanied by increased droplet size and fluorescence intensity (Fig. 1A–D). Fluorescence recovery after photobleaching (FRAP) experiments show efficient fluorescence recovery of mAIM2-DNA droplets after bleaching (Figs. 1E and S1E), demonstrating their dynamic nature and efficient molecular exchange with the external environment—a hallmark of typical liquid-liquid phase separation (LLPS). Consistent with mAIM2, hAIM2 is also induced by dsDNA to form prominent condensates *in vitro*, but with a stronger propensity, exhibiting gel-like condensation characteristics (Fig. S1F).

To better examine AIM2 condensate formation in cells, we generated HEK293T cell lines stably expressing GFP-tagged mAIM2 or hAIM2. Following transfection with cy5-labeled 100-bp dsDNA, both mAIM2 (Fig. 1F) and hAIM2 (Fig. S1G) readily form puncta with transfected dsDNA in the cytoplasm. FRAP experiments demonstrate robust fluorescence recovery of both mAIM2-DNA and hAIM2-DNA puncta (Figs. 1G and S1H), highlighting the dynamic and liquid-like features of AIM2-DNA condensates in cells. Taken together, these results show that DNA induces AIM2 to undergo liquid–liquid phase separation, leading to condensate formation both *in vitro* and in cells, a process conserved between human and mouse proteins.

We further investigated the conditions influencing AIM2 condensate formation. The phase diagram (Fig. S1I) shows that AIM2 forms condensates with dsDNA when the concentrations of AIM2 and dsDNA exceeds certain thresholds, with higher concentrations leading to more abundant and larger liquid droplets. Notably, AIM2 does not form robust condensates in the absence of dsDNA, even at relatively high protein concentrations, underscoring the essential role of DNA binding in this process (Fig. S1I). Increasing salt concentrations significantly weakens AIM2-DNA phase separation (Fig. S1D), indicating that ionic interactions between AIM2 and dsDNA are critical for condensate formation. In addition, the formation of AIM2–DNA droplets is largely inhibited by adding 5% 1,6-hexanediol (Fig. S1J). Furthermore, long dsDNA, which provides more binding sites, induces stronger AIM2 phase separation than short dsDNA (Fig. S1K), consistent with AIM2 activation requires a certain DNA length (Fig. S1L) and also suggesting the critical role of multivalent interactions in this process.

### Multivalent interactions drive AIM2-DNA liquid-phase condensation

**Figure 2.**
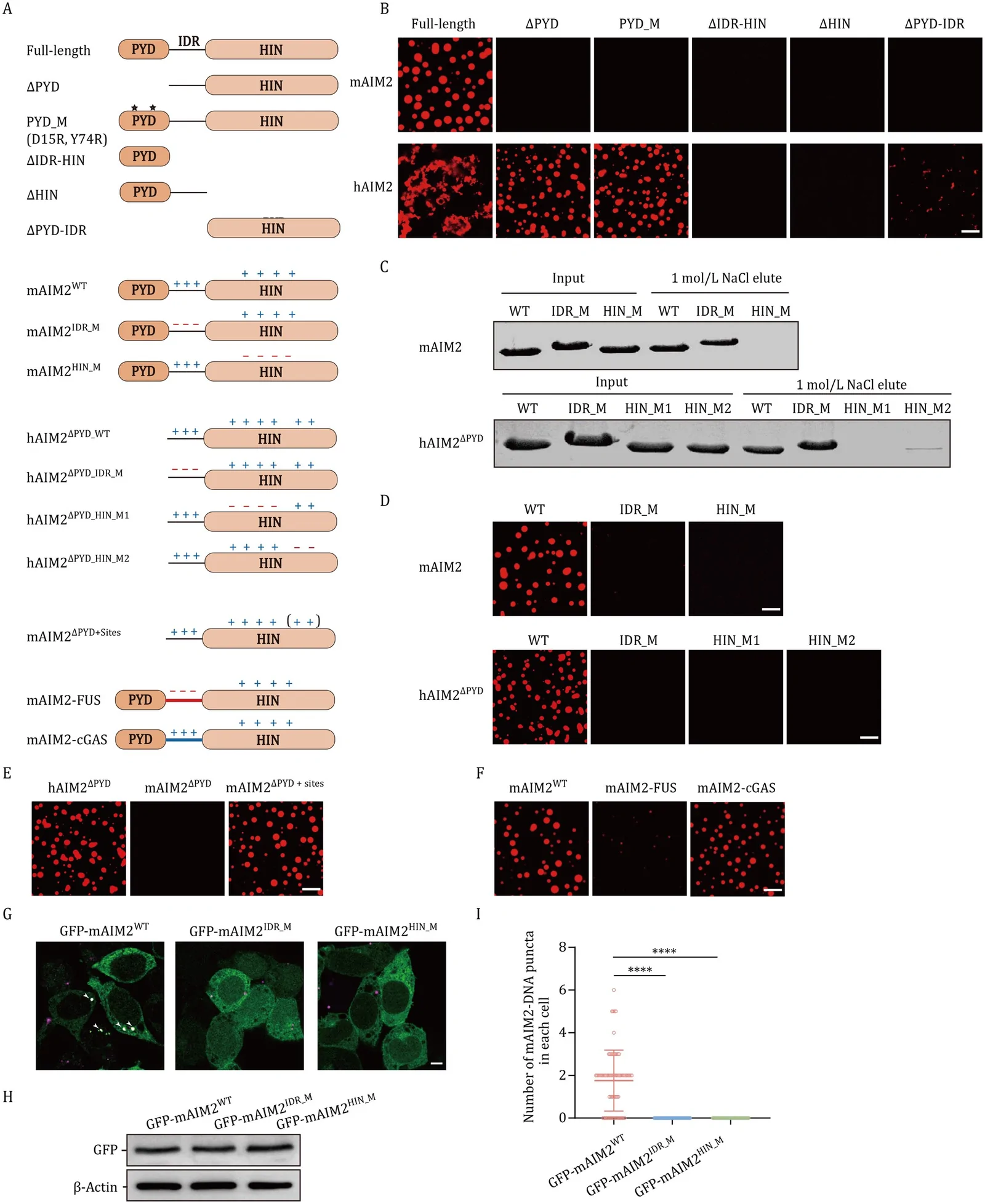
**Multivalent interactions drive AIM2-DNA liquid-phase condensation**. (A) Schematic of the designed AIM2 constructs. (B) Phase separation assay of indicated AIM2 constructs (5 μmol/L) with cy3-labeled 100-bp dsDNA (1 μmol/L) in 150 mmol/L NaCl buffer. Scale bar, 10 μm. (C) dsDNA cellulose beads pull-down assay of mAIM2^WT^ and its mutants (top panel), hAIM2^ΔPYD_WT^ and its mutants (bottom panel). (D) Phase separation assay of mAIM2^WT^ and its mutants (5 μmol/L) with cy3-labeled 100-bp dsDNA (1 μmol/L) in 300 mmol/L NaCl buffer (top panel). Phase separation assay of hAIM2^ΔPYD_WT^ and its mutants (5 μmol/L) with cy3-labeled 100-bp dsDNA (1 μmol/L) in 150 mmol/L NaCl buffer (bottom panel). Scale bar, 10 μm. (E) Phase separation assay of 5 μmol/L hAIM2^ΔPYD_WT^, mAIM2^ΔPYD_WT^ and mAIM2^ΔPYD+Sites^ with 1 μmol/L cy3-labeled 100-bp dsDNA in 150 mmol/L NaCl buffer. Scale bar, 10 μm. (F) Phase separation assay of 5 μmol/L mAIM2^WT^, mAIM2-FUS and mAIM2-cGAS with 1 μmol/L cy3-labled 100-bp dsDNA in 300 mmol/L NaCl buffer. Scale bar, 10 μm. (G) Representative images of mAIM2-DNA puncta (white) in HEK293T cells expressing GFP-mAIM2^WT^, GFP-mAIM2^IDR_M^ or GFP-mAIM2^HIN_M^ (green) at 5 h after transfecting with 1 μg/mL cy5-labeled 100-bp dsDNA (magenta) by using lipofectamine 3000 reagent and treating 25 µg/mL doxycycline to induce the expression of GFP-mAIM2^WT^ or its mutants. Arrowheads indicate AIM2-DNA puncta. Scale bar, 5 μm. (H) Immunoblot analysis of the expression of GFP-mAIM2^WT^ and its mutants in (G). (I) Quantification of the number of mAIM2-DNA puncta in each cell that formed as described in (G). *n* = 70. Data are mean ± SD. Statistical analyses were performed by using two-tailed Student’s t test. ****P < 0.0001.

To elucidate the molecular mechanisms of AIM2-DNA condensates formation, we performed *in vitro* phase separation assays using wild-type (WT) AIM2 and various truncation and mutation variants (Fig. 2A; Tables S1 and S2). Compared to WT protein, deletion of PYD (ΔPYD) in mAIM2 nearly abolishes its DNA-induced phase separation ability (Fig. 2B). Interestingly, because hAIM2 has a stronger condensation propensity than mAIM2 (Figs. 2B and S1F), PYD deletion does not completely eliminate its phase separation but shift it from a gel-like to a typical liquid-like state (Fig. 2B). We hypothesized that the essential role of PYD in AIM2-DNA phase separation could be attributed to homotypic PYD-PYD interactions, and indeed, mutating two key residues at the PYD-PYD interface (D15R and Y74R, referred to as PYD_M) produces a similar phenotype to the ΔPYD variant (Fig. 2B). Additionally, deleting the HIN domain (ΔHIN) or the entire IDR-HIN region (ΔIDR-HIN) completely abolishes DNA-induced phase separation in both mAIM2 and hAIM2, highlighting the critical role of the HIN domain (Fig. 2B). Furthermore, in hAIM2, deletion of the whole PYD-IDR region leads to a more pronounced reduction in phase separation than PYD deletion alone, emphasizing the IDR’s essential contribution. Collectively, these results demonstrate that all three major regions of AIM2—PYD, HIN, and IDR—are crucial for providing the multivalent interactions required for AIM2-DNA condensates formation.

To further understand how the HIN domain influences AIM2-DNA condensate formation, we generated a series of AIM2 mutants targeting potential multivalent interactions with dsDNA (Fig. 2A; Table S2). Structural analysis of AIM2^HIN^-DNA complex crystal structures, along with amino acid sequence examination, identifies several positively charged surface residues likely involved in DNA binding (Fig. S2A–C). Both mAIM2 and hAIM2 share a primary DNA binding site in a similar position, with slight differences in binding orientation (Fig. S2A–C), which has been considered as the assembly interface in the proposed AIM2 activation model (Jin et al., 2012; Ru et al., 2013). Interestingly, detailed analysis of the crystal packing lattice reveals two additional DNA binding sites in hAIM2 that are absent in mAIM2 (Fig. S2A–C). Charge-reversal substitutions at either the primary DNA binding sites (mAIM2^HIN_M^ and hAIM2^ΔPYD_HIN_M1^) or the additional DNA binding sites (hAIM2^ΔPYD_HIN_M2^) dramatically reduce DNA binding (Figs. 2C and Fig. S2A–C) and abolish phase condensation (Fig. 2D). We hypothesized that the stronger phase separation capacity of hAIM2 compared to mAIM2 may arise from these additional DNA binding sites. Indeed, introducing additional DNA binding sites into the PYD-deleted mAIM2 (mAIM2^ΔPYD+Sites^) restores a phase separation capacity comparable to that of its human counterpart (Fig. 2E). These findings show that both the primary and additional DNA binding sites are critical for DNA-induced phase separation and that the additional sites in hAIM2 enhance its condensation propensity relative to mAIM2.

Compared to the proposed AIM2 activation model suggesting that the IDR merely provides flexibility and length between PYD and HIN domains, our results reveal a previously unrecognized role of IDR in AIM2-DNA condensation (Fig. 2B). Detailed analysis reveals that both mAIM2 and hAIM2 IDRs harbor multiple positively charged residues (Fig. S2A), potentially promoting charge interactions critical for liquid-phase condensation. Charge-reversal mutations at these residues (mAIM2^IDR_M^ and hAIM2^ΔPYD_IDR_M^) practically abolish DNA-induced phase separation (Fig. 2D) while minimally affecting DNA binding (Figs. 2C and S2D). To further probe the role of IDR, we generated two chimeric proteins, mAIM2-cGAS and mAIM2-FUS, by replacing the mAIM2 IDR with an equally long, positively charged IDR from cGAS or negatively charged IDR from FUS, respectively (Fig. 2A). The AIM2-cGAS chimera retains phase separation similar to WT mAIM2, whereas the AIM2-FUS chimera shows a dramatic reduction (Fig. 2F). These data highlight the critical role of positively charged residues within the IDR in driving AIM2-DNA liquid-phase condensation.

To validate the importance of these sites in cells, we generated HEK293T cell lines expressing GFP-tagged WT mAIM2 and two condensation-deficient mutants targeting the IDR (mAIM2^IDR_M^) and HIN (mAIM2^HIN_M^) domains. Following transfection with cy5-labeled 100-bp dsDNA, cells expressing mutant AIM2 show no AIM2-DNA puncta formation, in contrast to the robust puncta observed in cells expressing WT protein (Fig. 2G–I). Taken together, both *in vitro* and cellular experiments demonstrate that reducing multivalent protein-DNA or protein–protein interactions weakens AIM2-DNA phase separation, whereas increasing interaction sites enhances its condensation. Moreover, these findings also highlight that, beyond the established PYD-PYD and primary HIN-DNA interfaces, previously underappreciated regions—such as the IDR and additional DNA binding sites within the HIN domain—are also critical for AIM2-DNA liquid-phase condensation.

### AIM2-DNA condensation facilitates inflammasome assembly

**Figure 3.**
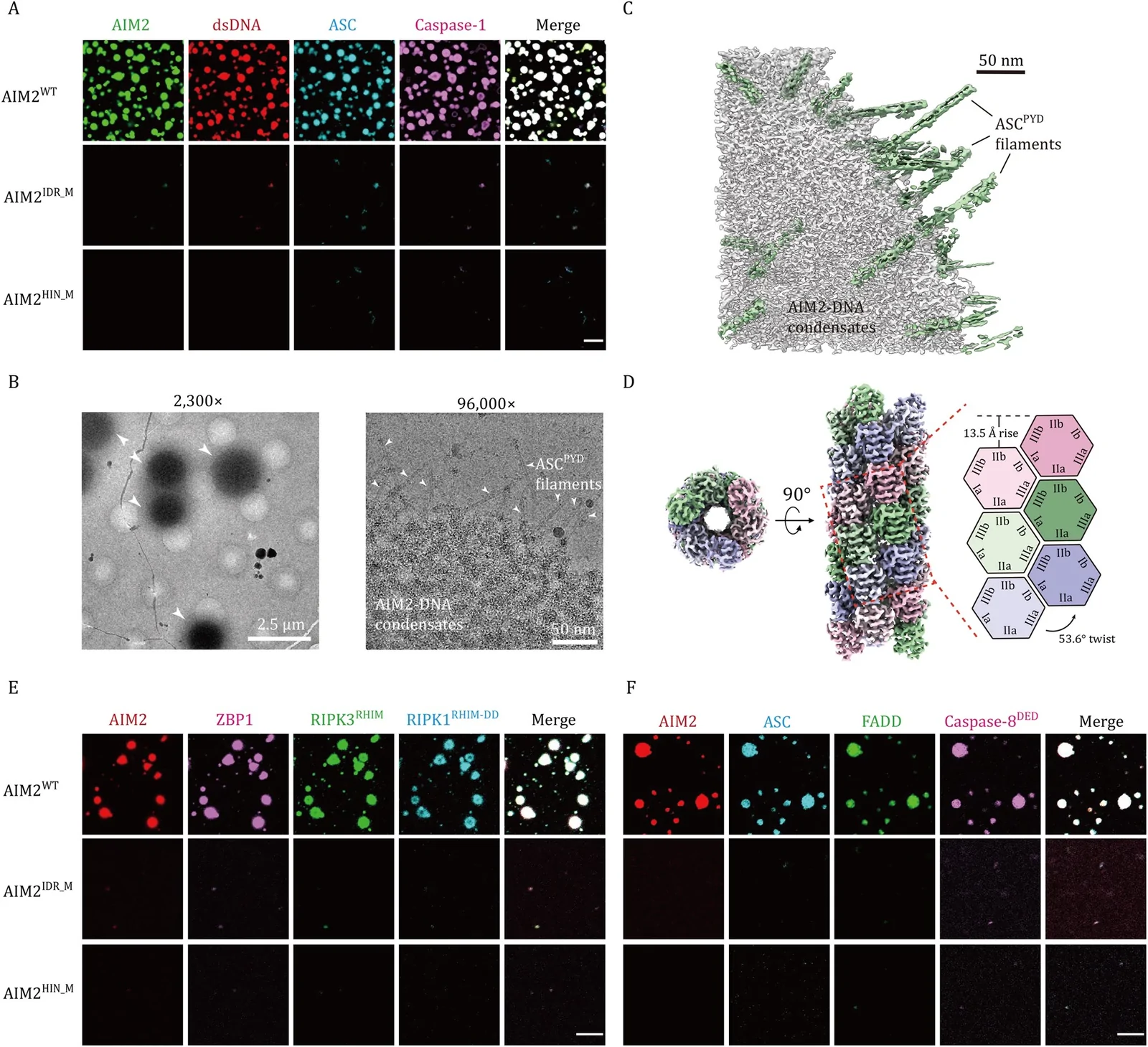
**AIM2-DNA condensation facilitates inflammasome and PANoptosome assembly**. (A) AIM2-DNA condensates enrich ASC and caspase-1. Incubation 2 μmol/L DyLight405-labeled ASC and 2 μmol/L AF647-labeled caspase-1(C284A) with condensates formed by 5 μmol/L AIM2^WT^ or its mutants and 1 μmol/L cy3-labled 100-bp dsDNA in 150 mmol/L NaCl buffer. Scale bar, 10 μm. (B) Cryo-EM images of AIM2-DNA-ASC^PYD^ sample at indicated magnification. Left panel: 2,300×, arrowheads indicate AIM2-DNA condensates. Scale bar, 2.5 μm. Right panel: 96,000×, arrowheads indicate the ASC^PYD^ filaments. Scale bar, 50 nm. (C) Cryo-ET of AIM2-DNA-ASC^PYD^ sample. Reconstructed tomogram with 3D rendering is shown, gray volume represents AIM2-DNA condensates, green volume represents ASC^PYD^ filaments. Scale bar, 50 nm. (D) Cryo-EM density map of the ASC^PYD^ filament from AIM2-DNA-ASC^PYD^ sample. Left panel: Top view. Middle panel: side view. Right panel: 2D schematic diagram of the ASC^PYD^ assembly. Three types of asymmetric interaction interfaces and helical indexing are shown. (E and F) AIM2-DNA condensates enrich multiple PANoptosome components. (E) Co-incubate 2 μmol/L AF647-labeled ZBP1, 2 μmol/L GFP-RIPK3^RHIM^, 2 μmol/L DyLight 405-labeled RIPK1^RHIM-DD^ with 5 μmol/L AF555-labeled AIM2 and 1 μmol/L 100-bp DNA in 150 mmol/L NaCl buffer. (F) Co-incubate 2 μmol/L DyLight 405-labeled ASC, 2 μmol/L AF488-labeled FADD, 2 μmol/L AF647-labeled caspase-8^DED^ with 5 μmol/L AF555-labeled AIM2 and 1 μmol/L 100-bp DNA in 150 mmol/L NaCl buffer. Scale bar, 5 μm.

Since dsDNA-activated AIM2 recruits ASC and caspase-1 to form the inflammasome, we hypothesized that DNA-induced AIM2 condensation may contribute to this assembly process. To test this, we incubated the purified ASC and an enzymatically inactive caspase-1^C284A^ with AIM2-DNA condensates. The results show that ASC is efficiently enriched within the condensates and promotes their solidification (Figs. 3A and S3A), while caspase-1 initially localizes to the periphery and then gradually infiltrates the condensates over time (Figs. 3A and S3B). In contrast, neither ASC nor caspase-1 is effectively concentrated when incubated with dsDNA and the condensation-deficient mutants (AIM2^IDR_M^ or AIM2^HIN_M^) (Fig. 3A). AIM2 phase condensation significantly increases its local concentration, likely promoting homotypic PYD-PYD interactions and the formation of ordered nucleation assemblies. The robust enrichment of ASC and caspase-1 in AIM2-DNA condensates further suggests a potential role in facilitating ASC filament formation and inflammasome assembly. Notably, compared with mAIM2, hAIM2 undergoes phase separation more rapidly, resulting in faster ASC recruitment and nucleation, as supported by the FRET-based kinetic analysis (Fig. S3C and S3D).

Given that ASC^PYD^ filament formation represents a hallmark and critical step of AIM2-mediated inflammasome activation, we intended to verify whether AIM2-DNA condensates promote this process. We generated cryo-electron tomography (cryo-ET) samples by incubating ASC^PYD^ with AIM2-DNA condensates and collected data by tilting the sample stage from –50° to +50°. The reconstructed tomogram shows that numerous ASC^PYD^ filaments extend from AIM2-DNA condensates (Fig. 3B and 3C; Video S1). Furthermore, to obtain high-resolution structural information, we performed cryo-electron microscopy (cryo-EM) analysis for these samples, obtaining a 2.7 Å reconstruction of the ASC^PYD^ filament (Figs. 3D and S3E–I, Table S3). The atomic model reveals a right-handed helical structure with a twist angle of 53.6° and an axial rise of 13.5 Å, mediated by three major interaction interfaces of each ASC^PYD^ subunit (Fig. 3D), consistent with previously reported activated ASC^PYD^ filament structures (Lu et al., 2014b). Together, these data show that DNA-induced AIM2 phase separation promotes inflammasome assembly by efficiently enriching and nucleating ASC and caspase-1.

### AIM2-DNA condensation facilitates PANoptosome assembly

In addition to mediating inflammasome assembly, AIM2 has also been shown to form PANoptosome with other proteins, including ZBP1, RIPK1, RIPK3, FADD, and caspase-8, to mediate inflammatory PANoptosis combining pyroptosis, apoptosis, and necroptosis (Lee et al., 2021; Malireddi et al., 2019; Oh et al., 2023; Sagulenko et al., 2013). To assess whether DNA-induced AIM2 phase separation facilitates PANoptosome assembly, we expressed and purified ASC, ZBP1, RIPK1^RHIM-DD^, RIPK3^RHIM^, FADD, and caspase-8^DED^, and simultaneously incubated them with dsDNA and either WT AIM2 or its condensation-deficient mutants. The results show that all these proteins are efficiently enriched into AIM2-DNA condensates (Fig. 3E and 3F), consistent with these proteins colocalizing with AIM2 puncta in cells, as a previous study reported (Lee et al., 2021). However, no effective enrichment was observed when these proteins were incubated with dsDNA and AIM2^IDR_M^ or AIM2^HIN_M^ mutants (Fig. 3E and 3F). These molecular evidences align well with prior cellular colocalization experiments and mechanistically explain why AIM2 deficiency disrupts PANoptosis activation.

Overall, these findings demonstrate that AIM2-DNA condensation not only promotes inflammasome assembly but also contributes to PANoptosome assembly by recruiting and enriching associated factors.

### AIM2-DNA condensation promotes broad immune activation

**Figure 4.**
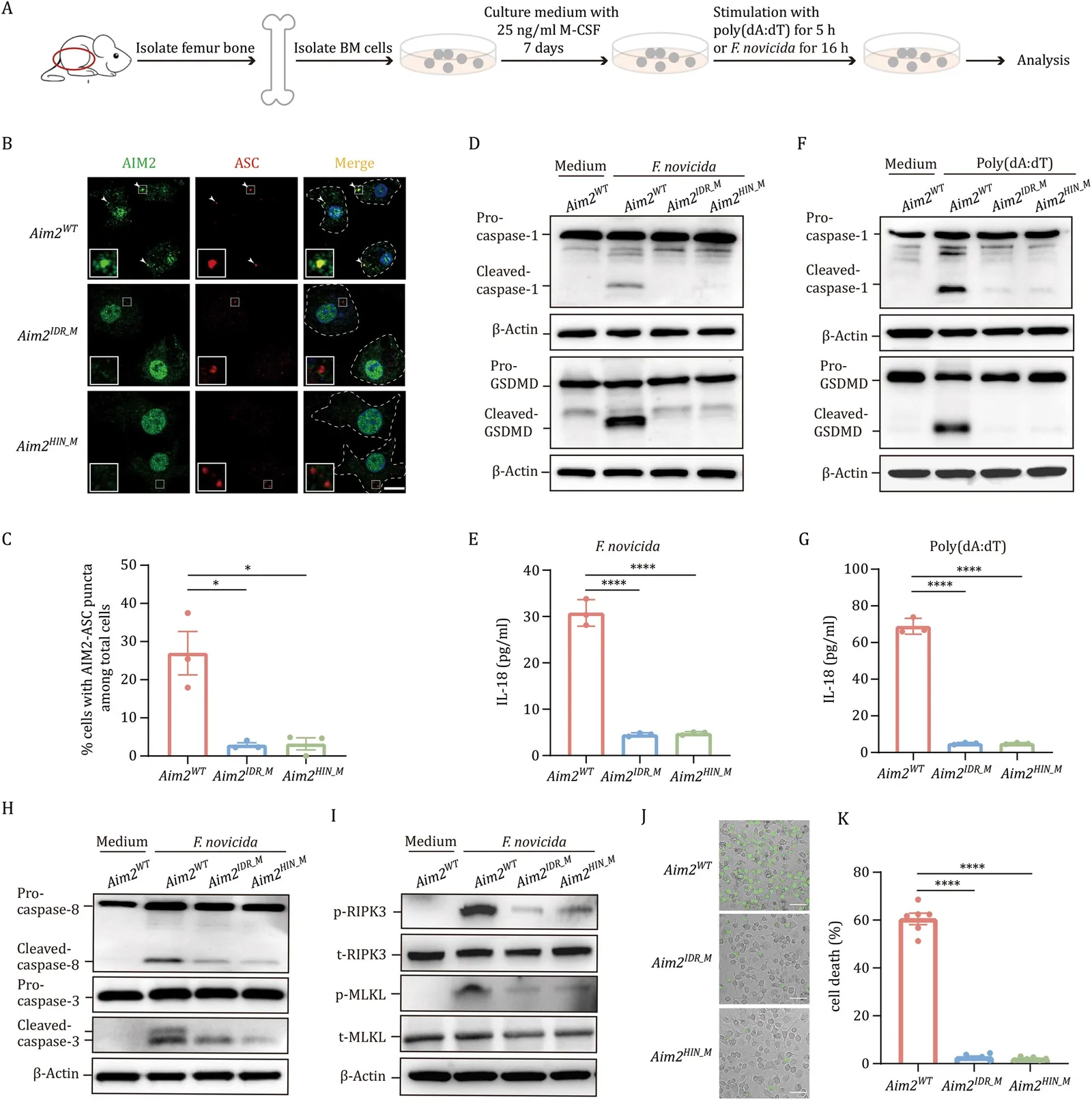
**AIM2-DNA condensation promotes broad immune activation**. (A) Schematic diagram of BMDMs was isolated from mice and cultured to perform the assay. (B) Immunofluorescence images of *Aim2^WT^*, *Aim2^IDR_M^*, and *Aim2^HIN_M^* BMDMs at 16 h after *F. novicida* infection. Arrowheads indicate the AIM2-ASC puncta. Scale bar, 10 μm. 4 μm for magnified images. (C) Quantification of the percentage of cells with AIM2-ASC puncta among all cells from (B). These data are representative of three independent experiments. Data are mean ± SEM. *Aim2^WT^*, *n* = 134; *Aim2^IDR_M^*, *n* = 130; *Aim2^HIN_M^*, *n* = 136. Statistical analyses were performed by using a two-tailed Student’s *t*-test. *P < 0.05. (D) Immunoblot analysis of pro- and cleaved-caspase-1, pro- and cleaved-GSDMD in *Aim2^WT^*, *Aim2^IDR_M^*, and *Aim2^HIN_M^* BMDMs at 16 h after *F. novicida* infection. (E) Cell supernatant IL-18 levels in *Aim2^WT^*, *Aim2^IDR_M^*, and *Aim2^HIN_M^* BMDMs at 16 h after *F. novicida* infection. These data from three independent experiments. Data are mean ± SEM. Statistical analyses were performed by using a two-tailed Student’s *t*-test. ****P < 0.0001. (F) Immunoblot analysis of pro- and cleaved-caspase-1, pro- and cleaved-GSDMD in *Aim2^WT^*, *Aim2^IDR_M^*, and *Aim2^HIN_M^* BMDMs at 5 h after stimulation with poly(dA: dT). (G) Cell supernatant IL-18 levels in *Aim2^WT^*, *Aim2^IDR_M^*, and *Aim2^HIN_M^* BMDMs at 5 h after stimulation with poly(dA: dT). These data from three independent experiments. Data are mean ± SEM. Statistical analyses were performed by using a two-tailed Student’s *t*-test. ****P < 0.0001. (H) Immunoblot analysis of pro- and cleaved-caspase-8, pro- and cleaved-caspase-3 in *Aim2^WT^*, *Aim2^IDR_M^*, and *Aim2^HIN_M^* BMDMs at 16 h after *F. novicida* infection. (I) Immunoblot analysis of phosphorylated RIPK3 (p-RIPK3), total RIPK3 (t-RIPK3), phosphorylated MLKL (p-MLKL), and total MLKL (t-MLKL) in *Aim2^WT^*, *Aim2^IDR_M^*, and *Aim2^HIN_M^* BMDMs at 16 h after *F. novicida* infection. (J) Cell death in *Aim2^WT^*, *Aim2^IDR_M^*, and *Aim2^HIN_M^* BMDMs at 16 h after *F. novicida* infection. Cell death was measured by the SYTOX Green uptake assay. Green indicates dead cells. Scale bar, 50 μm. (K) Quantification of cell death from (J). These data from three independent experiments. Data are mean ± SEM. Statistical analyses were performed by using a two-tailed Student’s *t*-test. ****P < 0.0001.

Since AIM2-DNA phase separation drives the assembly of both inflammasome and PANoptosome, we reasoned that this condensation process may also play a critical role in downstream immune activation across multiple cell death pathways. To verify this hypothesis, we generated transgenic mice harboring either *Aim2^WT^* or the condensation-deficient mutants (*Aim2^IDR_M^* or *Aim2^HIN_M^*) using the CRISPR-Cas9 method (Fig. S4A and S4B). Bone marrow-derived macrophages (BMDMs) isolated from these mice were used for inflammasome and PANoptosome activation assays (Fig. 4A). Upon infection with *Francisella novicida* (*F. novicida*), a Gram-negative bacterium known to replicate in the cytoplasm and activate AIM2 (Fernandes-Alnemri et al., 2010; Jones et al., 2010; Rathinam et al., 2010), *Aim2^WT^* BMDMs exhibit significantly more AIM2-ASC coenriched puncta compared to *Aim2^IDR_M^* or *Aim2^HIN_M^* BMDMs (Fig. 4B and 4C). Correspondingly, *Aim2^WT^* BMDMs show robust caspase-1 and GSDMD cleavage and high IL-18 release, whereas these activation markers are markedly reduced in the mutant BMDMs (Fig. 4D and 4E). In line with *F. novicida* infection, similar results were observed following poly(dA: dT) stimulation (Fig. 4F and 4G). In addition, we assessed apoptosis and necroptosis activation by evaluating caspase-8 and caspase-3 cleavage, as well as RIPK3 and MLKL phosphorylation, in BMDMs infected with *F. novicida*. Compared to *Aim2^WT^* BMDMs, the activation of these apoptosis- and necroptosis-associated proteins is significantly diminished in *Aim2^IDR_M^* and *Aim2^HIN_M^* BMDMs (Fig. 4H and 4I). Consistent with these activation markers, the overall cell death induced by *F. novicida* infection in *Aim2^WT^* BMDMs is dramatically higher than in the mutant groups (Fig. 4J and 4K). Taken together, these findings demonstrate that AIM2-DNA condensation is not only critical for the efficient assembly of inflammasome and PANoptosome but also plays an essential role in immune activation across pyroptotic, apoptotic, and necroptotic pathways.

### AIM2-DNA condensation promotes *in vivo* anti-infection defense

**Figure 5.**
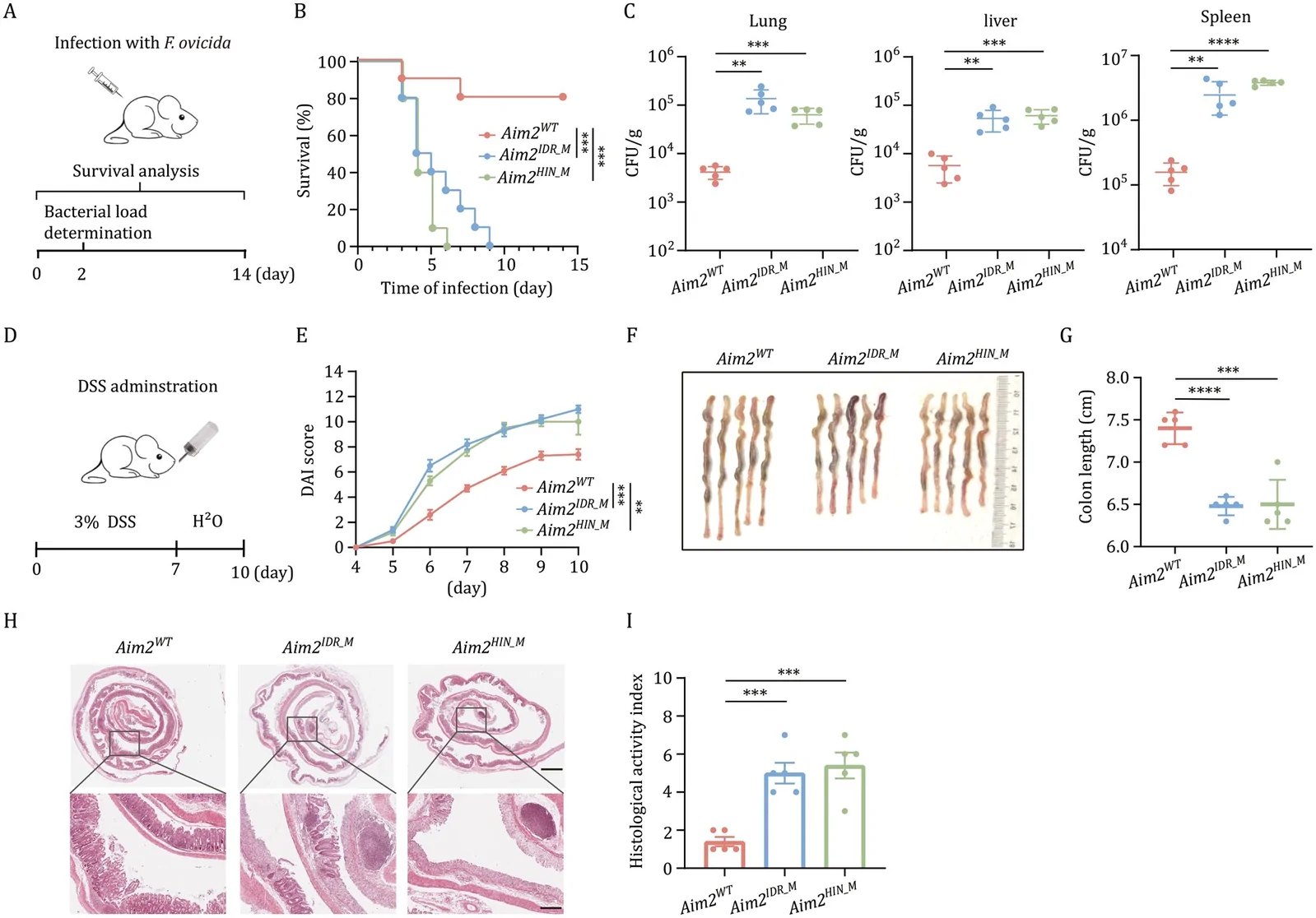
**AIM2-DNA condensation promotes *in vivo* anti-infection defense and maintains intestinal homeostasis**. (A) Schematic diagram of the mouse infection assay. (B) Survival of *Aim2^WT^*, *Aim2^IDR_M^*, and *Aim2^HIN_M^* mice (10 mice per group) infected with 5 × 10^5^ CFU of *F. novicida* per mouse. Statistical analyses were performed by using the log-rank (Mantel-Cox) test. ***P < 0.001. (C) Bacterial load in lung, liver, spleen obtained from *Aim2^WT^*, *Aim2^IDR_M^*, and *Aim2^HIN_M^* mice at 48 h after infection with 5 × 10^5^ CFU of *F. novicida* per mouse. Data are mean ± SEM, *n* = 5 mice per group. Statistical analyses were performed by using a two-tailed Student’s *t*-test. ****P < 0.0001, ***P < 0.001, **P < 0.01. (D) Schematic diagram of DSS administration assay. (E) The DAI score was evaluated daily after DSS administration. Data are mean ± SEM, *n* = 5 mice per group. Statistical analyses were performed by using two-way ANOVA. ***P < 0.001, **P < 0.01. (F) Pictures of the colon of *Aim2^WT^*, *Aim2^IDR_M^*, and *Aim2^HIN_M^* mice at day 10 after DSS administration. (G) The length of the colon in (F). Data are mean ± SEM, *n* = 5 mice per group. Statistical analyses were performed by using a two-tailed Student’s *t*-test. ****P < 0.0001, ***P < 0.001. (H) Representative H&E images of the Swiss roll intestine. scale bar, 1.5 mm. The representative region highlighted by the black box is magnified. scale bar, 250 μm. (I) Histological activity index in (H) was evaluated. Data are mean ± SEM, *n* = 5 mice per group. Statistical analyses were performed by using a two-tailed Student’s *t*-test. ***P < 0.001.

Given the essential role of AIM2-DNA condensation in activating multiple cell death pathways (Fig. 4) and the established link between AIM2 and infection defense (Fernandes-Alnemri et al., 2010; Lee et al., 2021; Rathinam et al., 2010), we hypothesized that this condensation process is crucial for *in vivo* protection against pathogen infection. To test this, we infected *Aim2^WT^* mice and condensation-deficient mutant mice (*Aim2^IDR_M^* and *Aim2^HIN_M^*) with *F. novicida* and monitored body weight changes, survival, and bacterial loads (Fig. 5A). Infection with *F. novicida* results in significantly higher mortality in *Aim2^IDR_M^* and *Aim2^HIN_M^* mice compared to *Aim2^WT^* mice (Fig. 5B). All *Aim2^HIN_M^* mice succumbed within 6 days, and all *Aim2^IDR_M^* mice died within 9 days, whereas 80% of *Aim2^WT^* mice survived beyond 14 days post-infection (Fig. 5B). Correspondingly, mutant mice exhibited more severe weight loss than *Aim2^WT^* mice (Fig. S4C). While *Aim2^WT^* mice regained body weight after an initial decline, mutant mice failed to recover (Fig. S4C). Consistently, bacterial loads in the lung, spleen, and liver of *Aim2^IDR_M^* and *Aim2^HIN_M^* mice are significantly higher than those in *Aim2^WT^* mice at 48 h post-infection (Fig. 5C). Together, these data demonstrate that the loss of AIM2-DNA condensation compromises host defense, highlighting its indispensable role in mounting an effective immune response against infection.

### AIM2-DNA condensation maintains intestinal homeostasis

AIM2 has also been implicated in maintaining intestinal homeostasis by mediating epithelial antimicrobial host defense (Hu et al., 2015; Ratsimandresy et al., 2017). To investigate whether AIM2-DNA phase separation contributes to this process, we established a DSS (dextran sodium sulfate)-induced acute colitis model and evaluated its effects in WT and mutant mice (Fig. 5D). Compared to *Aim2^WT^* mice, *Aim2^IDR_M^* and *Aim2^HIN_M^* mice exhibit markedly higher Disease Activity Index (DAI) scores following DSS administration (Fig. 5E). In addition, *Aim2^IDR_M^* and *Aim2^HIN_M^* mice show more severe colonic shortening than *Aim2^WT^* mice at day 10 post-DSS treatment (Fig. 5F and 5G). Histological assessment further reveals extensive crypt and goblet cell loss, along with increased inflammation infiltration in *Aim2^IDR_M^* and *Aim2^HIN_M^* mice compared to *Aim2^WT^* mice (Fig. 5H and 5I). These results indicate that AIM2 condensation deficiency aggravates colitis severity upon DSS administration, underscoring the critical role of DNA-induced AIM2 phase separation in maintaining intestinal homeostasis.

### AIM2-DNA condensation is modulated by host and pathogen factors

**Figure 6.**
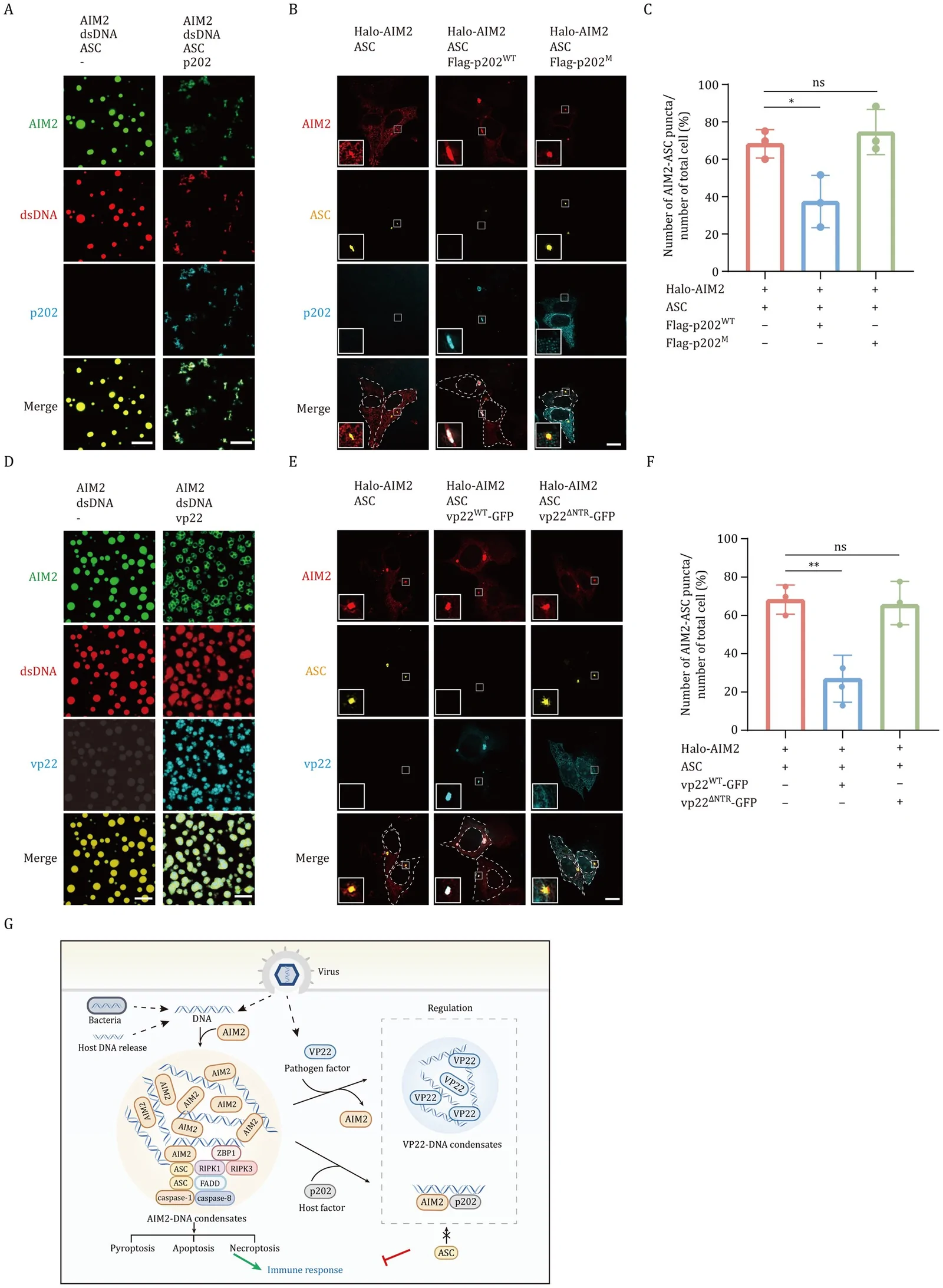
**Host- and pathogen-mediated regulation of AIM2-DNA phase separation**. (A) Images of 5 μmol/L AF488-labeled AIM2, 1 μmol/L cy3-labeled 100-bp dsDNA incubated with buffer (left panel) or 10 μmol/L Dylight 405-labeled p202 (right panel) in 150 mmol/L NaCl buffer. Scale bar, 10 μm. (B) Immunofluorescence images of HEK293T cells transfected with the indicated plasmids for 12 h. Scale bar, 10 μm. 5 μm for magnified images. (C) Quantification of AIM2-ASC puncta among total cells in (B). Data are mean ± SD. Statistical analyses were performed using a two-tailed Student’s *t*-test. *P < 0.05. ns, not significant. (D) Images of 5 μmol/L AF488-labeled AIM2 and 1 μmol/L cy3-labeled 100-bp dsDNA incubated with buffer (left panel) or 10 μmol/L AF647-labeled VP22 (right panel) in 150 mmol/L NaCl buffer. Scale bar, 10 μm. (E) Immunofluorescence images of HEK293T cells transfected with the indicated plasmids for 12 h. Scale bar, 10 μm. 5 μm for magnified images. (F) Quantification of AIM2-ASC puncta among total cells in (E). Data are mean ± SD. Statistical analyses were performed using a two-tailed Student’s *t*-test. **P < 0.01. ns, not significant. (G) Model of AIM2-DNA liquid-phase condensation and its function in immune activation and regulation.

Given the critical role of DNA-induced AIM2 condensation in immune activation and various *in vivo* functions, this process is likely a key regulatory target for different host and pathogen factors. p202, a mouse-encoded AIM2-like protein containing two HIN domains but lacking a PYD, has been reported to interact with AIM2 and suppress its activation (Roberts et al., 2009; Ru et al., 2013; Yin et al., 2013). To determine whether p202 influences AIM2-DNA phase separation, we incubated purified p202 with AIM2 and dsDNA. Compared to the buffer control, p202 significantly disrupts AIM2-DNA condensates, instead forming a few residual irregular aggregates with AIM2 and dsDNA (Fig. 6A). Consistently, in HEK293T cells co-expressing AIM2, ASC, and p202, the formation of AIM2-ASC coenriched puncta is markedly reduced compared to cells lacking p202 expression (Figs. 6B, 6C and S5A). Although p202 co-localizes with AIM2—mirroring the *in vitro* observations—ASC is not effectively recruited to AIM2 condensates (Fig. 6B). Furthermore, replacing WT p202 with a DNA-binding-deficient mutant (p202^M^) restores the formation of AIM2-ASC coenriched puncta (Figs. 6B, 6C and S5A). These findings indicate that p202 disrupts AIM2-DNA phase separation in a DNA-binding-dependent manner, thereby suppressing AIM2 activation. However, other cytosolic DNA-binding proteins such as cGAS and ZCCHC3 do not perturb AIM2 condensation (Fig. S5B and S5C), suggesting that p202-mediated disruption likely requires not only DNA binding but also a direct interaction with AIM2.

VP22, a tegument protein from HSV-1, has also been reported to inhibit AIM2 inflammasome activation by preventing AIM2 oligomerization (Maruzuru et al., 2018, 2021). To assess whether VP22 directly interacts with AIM2, we performed *in vitro* pull-down assays using purified proteins, with contaminating nucleic acids removed, but detected no clear interaction under physiological buffer conditions (Fig. S5D). We next examined whether VP22 interferes with AIM2-DNA phase separation. Co-incubation of VP22, AIM2, and dsDNA shows that VP22 robustly occupies a large portion of the AIM2-DNA condensates, forming its own phase separation with dsDNA while excluding AIM2 (Fig. 6D). Furthermore, when VP22 first forms condensates with DNA prior to AIM2 addition, AIM2 is sequestered to the periphery of VP22-DNA condensates (Fig. S5E). In both cases, AIM2-DNA phase separation is notably reduced. To validate these results in cells, we transfected HEK293T cells with plasmids that express AIM2, ASC, and VP22. Compared to cells lacking VP22, those expressing VP22 exhibit a significant reduction in AIM2-ASC puncta formation (Figs. 6E, 6F and S5G). Although AIM2 and VP22 initially colocalize at 12 h post-transfection (Fig. 6E), they exhibit distinct spatial separation by 24 h (Fig. S5F). Importantly, at both time points, ASC fails to be recruited to AIM2 foci (Figs. 6E and S5F), suggesting that VP22 effectively disrupts AIM2-mediated inflammasome assembly. To further delineate the role of VP22 phase separation, we employed VP22^ΔNTR^, a phase separation-defective mutant (Xu et al., 2021). Expression of VP22^ΔNTR^ restores AIM2-ASC puncta formation (Figs. 6E, 6F and S5G), indicating that VP22 relies on its phase separation ability to disrupt AIM2-DNA condensates, thereby inhibiting AIM2 activation.

## Discussion

Our study identifies a previously unrecognized mechanism of AIM2-mediated immune activation, wherein aberrant cytosolic dsDNA from infection or cellular stress induces AIM2 liquid–liquid phase separation to form biomolecular condensates (Fig. 6G). These condensates act as dynamic multifunctional platforms, assembling the AIM2 inflammasome by recruiting ASC and caspase-1, while also integrating ZBP1, RIPK1, RIPK3, FADD, and caspase-8 to form AIM2 PANoptosome, thereby triggering pyroptosis, apoptosis, and necroptosis. In addition, AIM2 phase separation may serve as a “checkpoint”—enabling rapid immune activation when cytosolic DNA reaches a physiologically meaningful length threshold while preventing inadvertent activation by shorter DNA fragments. Beyond its role in immune activation, AIM2-DNA condensates also serve as key regulatory hubs targeted by host- (e.g., p202) and pathogen-derived (e.g., VP22) factors, balancing immune homeostasis or facilitating immune evasion. *In vivo* experiments further underscore the essential role of AIM2-DNA condensation in mounting effective antimicrobial defenses and maintaining intestinal homeostasis. Notably, condensation-deficient AIM2 mutants fail to assemble immune complexes, initiate cell death pathways, or exert protective functions *in vivo*, highlighting the physiological importance of this phase separation process in innate immunity.

Additionally, our investigation into the molecular basis of AIM2-DNA condensation reveals that this process is driven by multivalent protein-DNA and protein–protein interactions, with the PYD, HIN, and IDR domains each being indispensable for condensate formation. PYD-PYD homotypic interactions not only promote phase separation but also facilitate the hierarchical assembly of PYD-dependent immune complexes, positioning condensates as platforms for signal amplification. The HIN domain, beyond its primary DNA-binding site, contains two additional DNA-binding sites in hAIM2 compared to its murine counterpart, enhancing DNA avidity and phase separation. This species-specific divergence likely optimizes innate immune responses by enabling cooperative DNA engagement and stabilizing higher-order AIM2-DNA assemblies. Meanwhile, the IDR, previously considered merely a flexible linker, emerges as a key modulator of condensate formation. Disrupting its charge distribution impairs phase separation without affecting DNA binding, suggesting it mediates weak multivalent interactions crucial for condensate assembly while preserving DNA recognition. Notably, IDR mutations that selectively disrupt phase separation severely compromise immune activation, infection resistance, and intestinal homeostasis, highlighting the instructive and causal role of phase separation in AIM2 signaling.

The currently identified AIM2-DNA condensation mechanism offers a more comprehensive understanding of AIM2-mediated immune activation compared to previously predicted models. Phase separation allows AIM2 to efficiently concentrate at dsDNA within the vast cytosolic space, addressing the challenge of how relatively low AIM2 levels converge on DNA targets. This process not only ensures DNA recognition but also clarifies the previously unresolved DNA length requirement for AIM2 activation. Furthermore, phase separation significantly increases the local concentration of AIM2 and DNA, enhancing the recruitment of downstream immune factors and promoting inflammasome and PANoptosome assembly. Additionally, previously overlooked sites within the IDR and HIN domains have been identified as critical for both phase separation and AIM2 function, providing new insights into how AIM2 interacts with DNA and assembles immune complexes. Moreover, this model uncovers a novel immune regulatory mechanism, where both host and viral proteins can modulate the condensation process. Notably, the viral protein VP22, despite not having evolved strong direct interactions with AIM2, can still fine-tune immune activation by influencing AIM2-DNA phase separation. This mechanism mirrors the regulatory role of this protein family in modulating cGAS-DNA phase separation (Bhowmik et al., 2021; Xu et al., 2021).

As another key cytosolic DNA sensor, cGAS has also been shown to undergo phase separation with DNA (Du and Chen, 2018). This raises the question of how AIM2 and cGAS behave when aberrant DNA accumulates in the same cellular environment. To explore this, we examined their subcellular localization following DNA stimulation in BMDMs and THP1 cells, both of which express endogenous AIM2 and cGAS. We observed that AIM2 and cGAS co-localize with DNA and form condensates (Fig. S5H). Consistent with the cellular results, cGAS also co-partitions with AIM2-DNA condensates *in vitro* (Fig. S5C). These observations suggest that the two sensors can coexist within shared DNA-rich condensates, although whether they influence each other’s condensation dynamics or downstream signaling remains an open question for future investigation.

In summary, this study uncovers a novel mechanism of AIM2-DNA condensation in immune activation and regulation, offering new perspectives on the physiological and pathological functions of AIM2, and providing potential therapeutic opportunities for targeting the condensation process to modulate immune responses.

## Supplementary Material

pwag024_Supplementary_Data

## Data Availability

The atomic coordinate of ASC^PYD^ filament has been deposited at the Protein Data Bank under accession numbers 9U8K. The cryo-EM density map has been deposited at the Electron Microscopy Data Bank under accession numbers EMD-63955. All other data supporting the results can be found in this paper. All other relevant data and material can be obtained from the corresponding authors upon request.

## Code availability

ImageJ was used for image analysis. GraphPad Prism was used for statistical analysis. CryoSPARC, SerialEM software, Warp, IMOD software package, MotionCor2, UCSF Chimera, UCSF Chimera X, Coot, PHENIX and Pymol were used for data collection and processing of cryo-ET and cryo-EM.

## Abbreviations

AIM2: absent in melanoma 2
bp: base pairs
BMDMs: bone marrow-derived macrophages
cryo-ET: cryo-electron tomography
cryo-EM: cryo-electron microscopy
dsDNA: double-stranded DNA
DSS: dextran sodium sulfate
DAI: Disease Activity Index
FRAP: fluorescence recovery after photobleaching
*F. novicida*: *Francisella novicida*
GSDMD: gasdermin D
IDR: intrinsically disordered region
LLPS: liquid–liquid phase separation
PAMP or DAMP: pathogen- or damage-associated molecular pattern
PRRs: pattern recognition receptors
PYD: pyrin domain
