# Supplementary material for: DNA-triggered AIM2 condensation orchestrates immune activation and regulation
Source: Protein Cell. 2026 Mar 25;17(9):807–20. doi: 10.1093/procel/pwag024 (PMC13521274; doi:10.1093/procel/pwag024)
Supplement: Supplementary file 1 [file pwag024_supplementary_data.zip › Supplemental materials.docx]

**METHOD DETAILS**

**Mice**

All mice were bred and housed under specific pathogen-Free facilities at the Institute of Biophysics, Chinese Academy of Sciences. Mouse maintenance and procedures were conducted in accordance with the ethical guidelines for animal research and were approved by the Biomedical Research Ethics Committee of the Institute of Biophysics, Chinese Academy of Science (SYXK2023187). All mice were maintained with 5 mice per cage in a specific-pathogen free facility with a 12h:12h light:dark cycle at 21-23°C. The following mouse strains were used in this study: *Aim2^WT^*, *Aim2^IDR_M^* and *Aim2^HIN_M^* mice on the C57BL/6 background were obtained from GemPharmatech corporation.

**Mammalian cell lines**

BMDMs and HEK293T were cultured in DMEM media (GBICO) supplemented with 10% fetal bovine serum (Thermo Fisher Scientific) at 37°C and 5% CO2. For BMDMs isolation and culture, primary murine bone marrow cells were flushed from tibias and femurs with chilled DMEM and cultured for 7 days in DMEM (C11885500BT, Gibco) supplemented with 10% FBS and 25 ng/ml M-CSF (MCE) to generate BMDMs. For doxycycline-inducible HEK293T stable cell lines, 10% Tet-System Approved FBS (Biological Industries) was added to the DMEM media instead of fetal bovine serum. THP-1 cells were cultured in RPMI media (GBICO) supplemented with 10% fetal bovine serum (Thermo Fisher Scientific) at 37°C and 5% CO2. Before stimulation, THP-1 cells were differentiated for 24 h with 1 μM PMA. All media was supplemented with 1% penicillin-streptomycin.

***Escherichia coli* strains**

*E.coli* BL21(DE3) cells (Tsingke) were used to express ASC, caspase-1, FADD, caspase-8^DED^, RIPK1^RHIM-DD^, RIPK3^RHIM^-GFP, ZBP1, VP22, p202, AIM2 and its mutants.

**Protein Expression and Purification**

*Aim2* and its constructs, *Asc*, *Caspase-1*, *Fadd*, *Caspase-8^DED^*, *Ripk1^RHIM-DD^* and *Ripk3^RHIM^-gfp* were inserted into pET28a plasmid with N-terminal TEV protease cleavable His6-MBP tag. *p202* was inserted into pET28a plasmid with C-terminal TEV protease cleavable His6-MBP tag. *vp22* was inserted into pRSFDUET-1 plasmid with N-terminal His6-SUMO tag. These proteins were expressed in BL21(DE3) cell strain. The cells were grown at 37°C until OD600 reached 0.8. The temperature was then shifted to 20°C and the cells were induced with 0.3 mM IPTG overnight. *Zbp1* was inserted into pFASTBAC plasmid with N-terminal TEV protease cleavable His6-MBP tag and was expressed in SF9 for 3 days. After cells were harvested by centrifugation and resuspended in buffer A (50 mM Tris-HCl pH 7.5, 500mM NaCl, 20 mM imidazole, 5 mM β-mercaptoethanol, 10% glycerol), cells were lysed by French Press and lysates were cleared by centrifugation. The supernatant was loaded onto a Ni-NTA affinity column (GE Healthcare). Sample was eluted with buffer B (50 mM Tris-HCl pH 7.5, 500mM NaCl, 500 mM imidazole, 5 mM β-mercaptoethanol, 10% glycerol). AIM2 and its constructs, p202, VP22 and ZBP1 were further applied to a 5-mL Hitrap Heparin column (GE Healthcare) with a linear NaCl gradient from 150 mM to 1 M. ASC, caspase-1 were further applied to a 5-mL Hitrap Q column (GE Healthcare) with a linear NaCl gradient from 150 mM to 1 M. Then samples were subjected to gel filtration on a Superdex G200 10/300 GL size-exclusion column (GE Healthcare) equilibrated with buffer C (20 mM Hepes pH 7.5, 300 mM NaCl, 1 mM DTT). The peak sample was concentrated, measured and stored at -80°C before use.

**Proteins Labeling, Phase Separation Assay and FRAP assay *in vitro***

Purified proteins were labeled with corresponding dyes by using Alexa Fluor™ Protein Labeling Kit (Thermo Fisher Scientific) according to the manufacturer’s protocols. Phase separation assay performed by mixing indicated proteins (2% dyes labeled), indicated dsDNA (2% cy3 labeled) and 0.2 mg/ml TEV, in a final volume of 50 ml at room temperature. Then mixing samples were loaded into 96-well plates (Corning) pre-coated with 20 mg/mL BSA (Sigma) to be imaged by a Nikon A1R+ confocal microscope with an oil immersion 60× objective lens. Concentration of salt, proteins and dsDNA are indicated in the Figure legends. For time-lapse imaging, images were acquired with 7 s interval in 60 min. For FRAP assay, the droplets were imaged with 7 s interval for 21 s and then photobleaching was performed with a 561 nm laser. After bleaching, the recovery images were acquired with 7 s interval in 10 min. The images and time-lapse movies were processed with ImageJ(Schindelin et al., 2012).

**dsDNA-coupled cellulose beads pull-down assay**

Proteins were incubated with dsDNA-coupled cellulose beads (Sigma) in the binding buffer (20 mM Tris-HCl pH 7.5, 150 mM NaCl), at 4°C for 1 h. After incubation, the beads were washed with the same binding buffer for three times. Then samples were eluted with buffer (20 mM Tris-HCl pH 7.5, 1 M NaCl). Input and eluted samples were analyzed by SDS-PAGE.

**Electromobility shift assays (EMSA)**

100ng FAM labeled -20bp dsDNA was incubated with 50-0.2 μM AIM2 protein in 20 μL binding buffer (20 mM Tris-HCl pH 7.5, 150 mM NaCl) at 4°C for 30 min. After incubation, samples were resolved on a native 6% PAGE gel in 0.5× TBE buffer at 4°C. Gels were then analyzed by fluorescence imaging systems. The band intensity was measured through ImageJ and K_D_ was calculated by GraphPad Prism 8.0 software.

**Fluorescence resonance energy transfer (FRET) assay**

ASC^PYD^ (106C) was labeled with sulfo-Cy3/Cy5 maleimide fluorophores in reaction buffer (20 mM HEPES pH 7.4, 150 mM NaCl, 1 mM TCEP). 1 μM AIM2 incubated with 0.5 μM DNA for 5 min before adding 2 μM ASC^PYD^ labeled with Cy3 and Cy5 fluorophores in reaction buffer (20 mM HEPES pH 7.4, 150 mM NaCl, 1 mM TCEP). Then immediately start recording the fluorescence signal. The data was processed and analyzed by GraphPad Prism 8.0 software.

**MBP pull-down assay**

MBP-AIM2 was incubated with Dextrin Sepharose beads (GE Healthcare) in the binding buffer (150mM NaCl, 20 mM Tris-HCl pH7.5, 1 mM DTT) at 4°C for 1 h and washed three times with the binding buffer. Excessive VP22 protein was incubated with the protein-coated beads in the binding buffer at 4°C for 1 h. Then beads were washed three times with the binding buffer. The samples were eluted by elution buffer (150mM NaCl, 20 mM Tris-HCl pH7.5, 1 mM DTT, 20 mM maltose). Input and eluted samples were analyzed by SDS-PAGE.

**Plasmids construction for cellular assays**

To generate the lentiviral vectors for doxycycline-inducible expression of AIM2 (pCW-GFP-AIM2), GFP-mAim2 and GFP-hAim2 fragments were amplified and inserted into pCW-Cas9-Blast vector (Addgene plasmid #83481). For the construction of transient expression plamids, Halo-mAim2, Flag-p202 and Vp22-sfgfp fragments were amplified and inserted into pCMV vector.

**Lentivirus packaging and Cell lines constructed**

For lentivirus packaging, pCW-GFP-AIM2, NRF packaging plasmid and VSV-G envelope plasmid were co-transfected to HEK293T cells. The lentivirus was harvested from the supernatant at 48 h post-transfection and filtered with a 0.45 μm polyvinylidene fluoride filter. Then HEK293T cells were infected with the lentivirus. After 72 h post-transfection, cells were treated with 25 μg/ml doxycycline to induce the expression of GFP-AIM2 and then GFP-positive cells were sorted by Flow Cytometer. Cells were cultured and the expression of GFP-AIM2 was analyzed by western blot.

**Imaging AIM2-DNA puncta and perform FRAP assay in cells**

The constructed HEK293T cells expressing GFP-AIM2 were plated onto collagen pre-coated coverslips. After 12 h post-plating, cells were induced by treating with 25 µg/mL doxycycline and transfected with 1 µg Cy5-labeled dsDNA oligoes for 5 h. Then imaging assay and FRAP assay were performed on a Zeiss LSM980 with Airyscan2 confocal laser scanning microscope equipped with a 63X /1.4 NA oil objective. The 488 nm laser were used for FRAP measurements. Bleaching was undertaken over a ~1 mm radius using 100% laser power, and the post-bleaching images were collected from a z-stack of 5 µm every 8 s (mAIM2-DNA puncta) or 5 s (hAIM2-DNA puncta). Fluorescence intensity was measured using Software ZEN (Blue edition 3.8), and the resulted intensity traces were normalized according to the procedures in easyFRAP-web.

For imaging of AIM2 puncta in BMDMs or THP-1, BMDMs or THP-1 were transfected with Cy5-lableled 100-bp dsDNA for 8 h or BMDMs were infected with *F. novicida* at a MOI of 50 for 16 h. Then cells were imaged after immunofluorescence staining. Immunofluorescence images were captured and analyzed using Zeiss LSM980 with Airyscan2 confocal laser scanning microscope equipped with a 63X /1.4 NA oil objective.

For imaging of the inhibition of AIM2-ASC puncta formation by p202 and VP22, HEK293T cells were transfected with indicated plasmids by using lipo3000 for 12 h or 20h. Then cells were imaged after staining with 30 nM of HaloTag-JF549 dye (Promega) at 37°C for 30 min and immunofluorescence staining. The cells were imaged using the Multi-SIM imaging system (Beijing Nanolnsights-tech Co., Ltd) with a 100X/1.49 NA oil objective (Nikon CFI SR HP Apo).

**Immunofluorescence**

The cells in immunofluorescence assays were fixed in 4% paraformaldehyde for 10 min, blocked with blocking buffer (3% normal donkey serum in PBST (PBS+0.1% Triton X-100) for 1 h, and incubated sequentially with primary antibodies including anti-AIM2 (1:200, Abcam, ab119791), anti-ASC (1:200, Santa Cruz Biotechnology, sc-271054), anti-Flag (1:200, Proteintech, 66008-4-Ig) diluted in blocking buffer at 4°C overnight. The specimen was washed twice with PBS and incubated with Alexa488/640 fluorochrome-conjugated secondary antibodies (1:1000, Invitrogen) diluted in blocking buffer for 1 h. Cells were then washed twice and finally imaged.

**Preparation of AIM2-DNA-ASC^PYD^ TEM grids**

AIM2-DNA-ASC^PYD^ sample preparation is performed by mixing 5 μM AIM2, 1 μM 100-bp dsDNA and 0.2 mg/mL TEV in 150 mM NaCl buffer. After incubation for 15 min, 5 μM ASC^PYD^ was added to the reaction solution. After 10 min, sample was loaded onto the R1.2/1.3 Cu 300 mesh grids (Quantifoil) and then plunge-frozen into liquid ethane by using a Vitrobot (Thermo Fisher Scientific). The grids were stored in liquid nitrogen.

**Data collection and processing of cryo-ET**

The prepared grids were subjected to a 300 kV Titan Krios microscope (FEI, Thermo Fisher Scientific), equipped with a post-column energy filter (Gatan) and a K2 Summit direct detector camera (Gatan). Using SerialEM software (Mastronarde, 2005), tilt-series were acquired from −50° to +50°, with 2° increments. Images were recorded in movie mode at 11 frames per second, with an object pixel size of 1.36 Å (magnification of 105,000x) and a defocus of -3 to -6 μm with a dose-symmetric tilt scheme using the beam-shift method (Wu et al., 2019). The dose was set at 2.5 e/Å2 per tilt image collected over frames.

Raw images of the tilt series were processed in Warp for motion correction (Tegunov and Cramer, 2019), contrast transfer function (CTF) estimation and tilt series sorting. Tilt series was aligned using the patch-tracking method included in the IMOD software package (Kremer et al., 1996). Then the alignment parameters were sent back to Warp, and the tomograms were reconstructed at 4× binning (voxel size = 5.44 Å).

For data processing, motion correction, CTF estimation, dose filtering and tilt-series sorting were performed in Warp. The dataset was processed with the denoising network in Warp for visualization purposes only.

**Data collection and processing of cryo-EM**

Data collection of cryo-EM was performed on a 300 kV Titan Krios electron microscope (FEI) equipped with K2. Summit camera (Gatan) and a GIF Quantum energy filter operated with a slit width of 20 eV. All cryo-EM super-resolution micrographs were collected automatically using the Serial-EM package, yielding an image stack with a pixel size of 0.52 Å. Images were recorded at a defocus range of -1.2 μm to -1.8 μm, with a total electron dose of 60 e/Å2 over 32 movie frames. Images were recorded by beam-image shift data collection methods (Mastronarde, 2005). Each movie stack was motion-corrected by MotionCor2 (Zheng et al., 2017). The dose-weighted micrographs were kept for further image processing using CryoSPARCv4.5.1 (Punjani et al., 2017).

The exact defocus value and contrast transfer function (CTF) of each micrograph were estimated using CryoSPARC’s patch CTF estimation tool. Particles were automatically picked using Blob picking and Templet picking. For the data of ASC^PYD^ filament, a total of 402,816 particles were auto-picked from 581 micrographs. After 2D classification, 81,574 particles with good features were kept for further data processing. A total of 45,400 particles were used for further homogeneous refinement and non-uniform refinement with C3 symmetry resulting in a 2.7 Å map, whose resolution was estimated based on the gold-standard Fourier shell correlation (FSC) with 0.143 criterion.

For the atomic model of ASC^PYD^ filament, use the structure of ASC^PYD^ filament predicted by AlphaFold-Multer (Evans et al., 2022), as the initial model, was fitted into the cryo-EM map using UCSF Chimera (Pettersen et al., 2004). The resulting model was then manually rebuilt in COOT (Emsley et al., 2010) and further refined by real space refinement in PHENIX (Adams et al., 2010). Structural Figureures were generated using PyMOL and ChimeraX (Goddard et al., 2018).

**BMDMs stimulation and infection**

BMDMs were plated onto 12-well plates at a density of 1 × 10^6^ cells per well and incubated overnight before use. Stimulation and infection assays were performed as described previously (Tweedell et al., 2020). For stimulation with poly(dA:dT), each well of BMDMs was transfected with 2 μg poly(dA:dT) by using Xfect reagent for 5 h. For *F. novicida* infection, *F. novicida* was grown in TSB supplemented with 0.2% L-cysteine overnight at 37 °C. Each well of BMDMs was infected with *F. novicida* at a MOI of 50 for 16 h. For cell death analysis, each well of BMDMs was added with 20 nM SYTOX and imaged by confocal at indicated time point.

***F. novicida* infected mice**

6- to 8-week-old *Aim2^WT^ mice*, *Aim2^IDR_M^* mice and *Aim2^HIN_M^* mice were used for infections. Mice were infected subcutaneously with 5 × 10^5^ CFU of *F. novicida* in 200 μl PBS. For survival analysis, infected mice were continuously observed for 12 days. To evaluate the bacterial load, the spleen, liver and lung tissues of infected mice were collected and homogenized to perform plaque assays.

**Experimental colitis and disease assessment**

Mice were administered DSS (3% w/vol; molecular weight: 36-50 kDa; MP Biomedicals) in drinking water for 7 days, followed by regular drinking water for 3 days. Clinical signs of colitis were evaluated based on the DAI score including body weight loss, occult blood, and stool consistency. In brief, the weight loss score was determined as follows: 0, no weight loss; 1, loss of 1-5% original weight; 2, loss of 6-10% original weight; 3, loss of 11-20% original weight; 4, loss of >20% original weight. The bleeding score was determined as follows: 0, no blood by using Haemoccult analysis; 1, positive Haemoccult; 2, visible blood traces in stool; and 3, gross rectal bleeding. The stool score was graded as follows: 0, well-formed pellets; 1, semiformed stools that did not adhere to the anus; 2, pasty semiformed stool that attached to the anus; and 3, liquid stools that attached to the anus.

For the histological activity index evaluation, harvested colon sections were stained with H&E. The histological activity index was assessed according to the loss of goblet cells and crypts and the extent of inflammatory cell infiltration. Crypt gland destruction was scored as follows: 0, normal; 1, a small amount of crypt loss; 2, a large amount of crypt loss; 3, extensive crypt loss. Goblet cells loss was scored as follows: 0, normal; 1, a small amount of goblet cells loss; 2, a large amount of goblet cells loss; 3, extensive goblet cells loss. Inflammatory cell infiltration was scored as follows: 0, normal; 1, infiltration around crypt glands; 2, infiltration of mucosal muscle layer; 3, extensive infiltration of the mucosal muscle layer with oedema; 4, infiltration of the submucosal layer.

**Immunoblot and ELISA analysis**

For caspase analysis, each well of BMDMs (contain 400 μL supernatant) was added with 100 μl 5× SDS loading buffer (15 g tris, 74 g glycine, 5 g SDS, 1× protease inhibitors, 1× phosphatase inhibitors in 1L ddH2O). Then combined supernatant and protein lysates were collected. For other analysis, the supernatants were removed and cells were washed with PBS, then each well of BMDMs was added with 80 μL RIPA lysis buffer (Solarbio) and collected. Collected samples were denatured at 95 °C for 10 min and then analyzed by gel electrophoresis. Protein was then wet transferred to a 0.45 μm PVDF membrane (Millipore) in ice-cold transfer buffer (25mM Tris, 192mM glycine, 20% methanol). The membrane was blocked with 5% skim milk in TBST for 1 h and then incubated with indicated primary antibodies overnight at 4°C : anti-AIM2 rabbit pAb (ABclonal, A25252,1:1000) , anti-ASC/TMS1 rabbit mAb (CST, 17507, 1:1000), anti-caspase-1 mouse mAb (AdipoGen, AG-20B-0042-C100, 1:1000), anti-GSDMD rabbit mAb (Abcam, ab209845, 1:1000), anti-cleaved caspase-8 rabbit mAb (CST, 8592, 1:1000), anti-caspase-3 rabbit mAb (CST, 14220, 1:1000), anti-cleaved caspase-3 rabbit pAb antibody (CST, 9661, 1:1000), anti-pMLKL rabbit mAb (CST, 37333, 1:1000), anti-pRIPK3 rabbit mAb (CST, 91702, 1:1000), anti-MLKL rabbit mAb (ABclonal, A21894), anti-RIPK3 rabbit mAb (CST, 95702, 1:1000), anti-β-actin mouse mAb (ABclonal, WH278304). After being incubated with primary antibody and washed with TBST, the membrane was incubated with appropriated second antibodies for 1 h: HRP-conjugated goat anti-rabbit IgG (ABclonal, AS014, 1:6000), anti-mouse IgG (ABclonal, AS003, 1:6000). After washing with TBST, sample were visualized by using SuperSignal West Atto Substrate and imaged by Tanon-5200 machine (Tanon).

For ELISA analysis, cell supernatant was collected and analyzed by ELISA kit (Beyotime, PI553) according to the manufacturer’s instructions.

**Statistical analysis**

GraphPad Prism 8.0 software was used to perform statistical analysis. Statistical significance was determined by two-tailed Student’s t test or one-way ANOVA. Detailed information is available in the Figureure legends.

**References**

Adams P D, Afonine P V, Bunkóczi G, et al. PHENIX: a comprehensive Python-based system for macromolecular structure solution. Acta Crystallogr D Biol Crystallogr 2010;**66**:213-221.

Emsley P, Lohkamp B, Scott W G, et al. Features and development of Coot. Acta Crystallogr D Biol Crystallogr 2010;**66**:486-501.

Evans R, O’Neill M, Pritzel A, et al. Protein complex prediction with AlphaFold-Multimer. bioRxiv 2022.

Goddard T D, Huang C C, Meng E C, et al. UCSF ChimeraX: Meeting modern challenges in visualization and analysis. Protein Sci 2018;**27**:14-25.

Kremer J R, Mastronarde D N & McIntosh J R. Computer visualization of three-dimensional image data using IMOD. J Struct Biol 1996;**116**:71-76.

Mastronarde D N. Automated electron microscope tomography using robust prediction of specimen movements. J Struct Biol 2005;**152**:36-51.

Pettersen E F, Goddard T D, Huang C C, et al. UCSF Chimera--a visualization system for exploratory research and analysis. J Comput Chem 2004;**25**:1605-1612.

Punjani A, Rubinstein J L, Fleet D J, et al. cryoSPARC: algorithms for rapid unsupervised cryo-EM structure determination. Nat Methods 2017;**14**:290-296.

Schindelin J, Arganda-Carreras I, Frise E, et al. Fiji: an open-source platform for biological-image analysis. Nat Methods 2012;**9**:676-682.

Tegunov D & Cramer P. Real-time cryo-electron microscopy data preprocessing with Warp. Nat Methods 2019;**16**:1146-1152.

Tweedell R E, Malireddi R K S & Kanneganti T D. A comprehensive guide to studying inflammasome activation and cell death. Nat Protoc 2020;**15**:3284-3333.

Wu C, Huang X, Cheng J, et al. High-quality, high-throughput cryo-electron microscopy data collection via beam tilt and astigmatism-free beam-image shift. J Struct Biol 2019;**208**:107396.

Zheng S Q, Palovcak E, Armache J P, et al. MotionCor2: anisotropic correction of beam-induced motion for improved cryo-electron microscopy. Nat Methods 2017;**14**:331-332.

**Supplemental Figures**

**Figure S1. dsDNA induces AIM2 to form liquid-phase condensation *in vitro* and in cells.** (A) The distribution of cancer-associated missense mutations in *Aim2*. Data are from the Cancer Genome Atlas (TCGA) database. (B) Immunofluorescence images of BMDMs and THP-1 at 8 h after transfecting with cy5-labeled 100-bp dsDNA. The AIM2-DNA puncta highlighted by the white box is magnified at the bottom left. Scale bar, 4 μm. 3 μm for magnified images. (C) Immunofluorescence images of BMDMs at 16 h after *F. novicida* infection. The AIM2-ASC puncta highlighted by the white box is magnified at the bottom left. Scale bar, 10 μm. 4 μm for magnified images. (D) Images of the condensates formed by 5 μM AF488-labeled mAIM2 and 1 μM 100-bp dsDNA in indicated salt concentration. Scale bar, 10 μm. (E) FRAP analysis of mAIM2-DNA droplets in 150 mM NaCl buffer or 300 mM NaCl buffer. These droplets were formed by incubating 5 μM AIM2 with 1 μM DNA. Values shown are means ± SD. n=3 condensates. (F) Images of the condensates formed by 5 μM AF488-labeled hAIM2 and 1 μM cy3-labeled 100-bp dsDNA in 300 mM NaCl buffer. Scale bar, 10 μm. (G) Representative live-cell images of hAIM2-dsDNA puncta in HEK293T cells expressing GFP-hAIM2 (Tet-on) at 5h after transfecting with 1 μg/mL cy5-labeled 100-bp dsDNA and treating 25 µg/mL doxycycline to induce the expression of GFP-hAIM2. The hAIM2-DNA puncta highlighted by the white box is magnified at the bottom left. scale bar, 4 μm; 3 μm for magnified images. (H) FRAP analysis of hAIM2-DNA puncta that shown in (G). Left panel: time-lapse images of hAIM2-DNA puncta before and after photobleaching. 3 μm for magnified images. Right panel: quantification of FRAP data. Values shown are means ± SD. n=3 puncta. (I) Phase separation diagram of AF488-labeled mAIM2 and cy3-labeled 100-bp dsDNA in 300 mM NaCl buffer. Merged images are shown. Scale bar, 10 μm. (J) Images of the condensates formed by 5 μM AF488-labeled mAIM2 and 1 μM DNA in 300 mM NaCl buffer after adding buffer (left panel) or 5% 1,6-hexanediol (right panel). (K) Images of the condensates formed by 5 μM AF488-labeled mAIM2 and 1 μM indicated length DNA in 300 mM NaCl buffer. Scale bar, 10 μm. (L) Immunoblot analysis of pro- and cleaved-caspase-1 in BMDMs at 5 h after stimulation with 2 μg indicated length DNA.

**Figure S2. The analysis of amino acid sequences, protein structures, surface potential and mutants of AIM2.** (A) Sequences of hAIM2 and mAIM2 were aligned and analyzed by Jalview. Domain elements of AIM2 are indicated above the sequence alignment. Conserved residues are shaded in yellow. Positively charged residues within IDR are in purple boxes. Main DNA binding sites are in blue boxes and additional DNA binding sites are in green boxes. (B) Left panel: crystal structure of hAIM2^HIN^:dsDNA (PDB: 3RN2). Right panel: crystal structure of mAIM2^HIN^:dsDNA (PDB: 4JBM). Main DNA binding sites are shown in blue stick. Additional DNA binding sites are shown in green stick. (C) Left panel: surface potential of hAIM2^HIN^. Right panel: surface potential of mAIM2^HIN^. Positive charge is represented by blue and negative charge is represented by red. (D) Electrophoretic mobility shift assay (EMSA) of mAIM2^WT^ and its mutants (top panel), hAIM2^ΔPYD_WT^ and its mutants (bottom panel). KD shown are means ± SD, n=3.

**Figure S3. AIM2-DNA condensation facilitates inflammasome and PANoptosome assembly.** (A) FRAP analysis of AIM2–DNA condensates after the addition of ASC. (B) Time-lapse images of droplets that formed as described in Figure 3A. Scale bar, 5 μm. (C) Schematic diagram of FRET assay. ASC^PYD^ labeled with Cy3 and Cy5 fluorophores incubated with AIM2-DNA condensates. (D) FRET assay of AIM2-DNA condensates nucleate ASC^PYD^ filament formation. 1 μM AIM2 incubated with 0.5 μM DNA for 5 min before adding 2 μM ASC^PYD^ labeled with Cy3 and Cy5 fluorophores. Then immediately start recording the fluorescence signal. (E) Representative raw micrograph of ASC^PYD^ filament from AIM2-DNA- ASC^PYD^. (F) Selected 2D class averages of ASC^PYD^ filament. (G) Data processing flow chart. (H) Gold-standard FSC curves used for global-resolution estimates within cryoSPARC. (I) Local resolution distribution of ASC^PYD^ filament.

**Figure S4. The generation of *Aim2^WT^*, *Aim2^IDR_M^*, *Aim2^HIN_M^* mice and infection assay.** (A) Schematic of generate three types of transgenic mice (*Aim2^WT^*, *Aim2^IDR_M^* and *Aim2^HIN_M^* mice) by CRISPR-Cas9. (B) Immunoblot analysis of AIM2 expression in *Aim2^WT^*, *Aim2^IDR_M^* and *Aim2^HIN_M^* BMDMs after stimulation with *F. novicida* (left panel) or poly(dA:dT) (right panel). (C) Weight loss of *Aim2^WT^*, *Aim2^IDR_M^* and *Aim2^HIN_M^* mice after infection with 5×10^5^ CFU of *F. novicida.*

**Figure S5. Host- and pathogen-mediated regulation of AIM2-DNA phase separation.** (A) Immunoblot analysis of the expression of AIM2, ASC, p202 in Figure 6B. (B) Images of droplets formed after co-incubating 5 μM AF488-labeled AIM2, 10 μM AF647-labeled ZCCHC3 and 1 μM cy3-100bp dsDNA. (C) Images of droplets formed after co-incubating 5 μM AF488-labeled AIM2, 10 μM AF647-labeled cGAS and 1 μM cy3-100bp dsDNA. (D) MBP pull-down assay of MBP-AIM2 and VP22. (E) Images of 5 μM AF488-labeled AIM2 incubated with 1 μM cy3-labeled 100-bp dsDNA (left panel) or preformed VP22-DNA condensates that is formed by 10 μM AF647-labeled VP22WT and 1 μM cy3-labeled 100-bp dsDNA (right panel). Scale bar, 10 μm. (F) Immunofluorescence images of HEK293T cells transfected with indicated plasmids for 24 h. Scale bar, 10 μm. 5 μm for magnified images. (G) Immunoblot analysis of the expression of AIM2, ASC, VP22 in Figure 6E. (H) Immunofluorescence images of BMDMs and THP-1 at 8 h after transfecting with cy5-labeled 100-bp dsDNA. Arrowheads indicate AIM2-cGAS-DNA puncta. Scale bar, 5 μm.

**Video S1. Z-axis fly-through of the reconstructed tomogram showing the intact AIM2-DNA condensates. Arrowheads indicate ASC^PYD^ filaments.**

**Supplemental Tables**
